# Determination of equilibria constants of arginine:glycine amidinotransferase (AGAT)-catalyzed reactions using concentrations of circulating amino acids

**DOI:** 10.1007/s00726-022-03218-5

**Published:** 2022-12-07

**Authors:** Dimitrios Tsikas

**Affiliations:** Institute of Toxicology, Core Unit Proteomics, Hannover Medical School, Carl-Neuberg-Strasse 1, 30625 Hannover, Germany

**Keywords:** AGAT, Citrulline, Equilibrium, Guanidinoacetate, Homoarginine, Lysine, Metformin, Ornithine, Supplementation

## Abstract

Arginine:glycine amidinotransferase (AGAT) catalyzes mainly two reactions that generate 1) L-homoarginine (hArg) from L-arginine and L-lysine (*K*_harg_) and 2) guanidinoacetate (GAA) and L-ornithine from L-arginine and glycine (*K*_gaa_). Previously, we found that pharmacological treatment of Becker muscular dystrophy (BMD) patients with metformin or L-citrulline resulted in antidromic effects on serum hArg and GAA concentrations, seemingly acting as an inhibitor and effector of AGAT activity, respectively. Here, we used data of this study as a model to determine *K*_harg_ and *K*_gaa_ values by using the concentrations of the participating amino acids measured in serum samples of the BMD patients. The study aimed to prove the general utility of this approach to investigate effects of amino acids and drugs on AGAT-catalyzed reactions in vivo in humans.

## Introduction

**Scheme 1:**
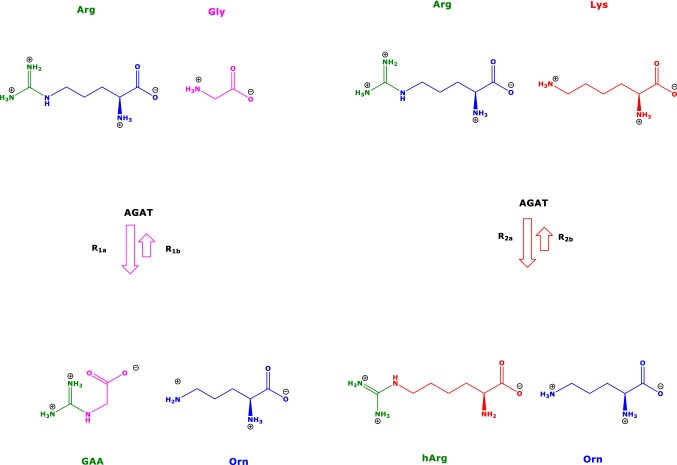
Simplified schematic of the two reactions catalyzed by L-arginine:glycine amidinotransferase (AGAT). The “forward” reactions are assumed to be preferred than the “reverse” reactions as symbolized by longer arrows

Amino acids are involved in numerous physiological pathways in all types of cell. They are distributed by the way of blood throughout the organism. Amino acids are rapidly inter-changed between extra- and intracellular spaces by specific amino acid transporters (Gauthier-Coles et al. 2021). Besides their involvement in protein synthesis, amino acids are substrates of numerous metabolic and catabolic enzymes. One of these enzymes is the two-substrates/two-products arginine:glycine amidinotransferase (AGAT; EC 2.1.4.1). AGAT mainly catalyzes the reaction of L-arginine (Arg) with glycine (Gly) to form guanidinoacetate (GAA) and L-ornithine (Orn) (R1) (Scheme 1). AGAT also catalyzes the reaction of Arg with L-lysine (Lys) to form L-homoarginine (hArg) and Orn (R2) (Tsikas and Wu 2015; Tsikas 2022). In these reactions, Arg, hArg and GAA are the amidine (NH_2_-C^+^ = NH) group donors; Gly and Orn are the acceptors of the donated amidine groups via their *α*- and *δ*-amine groups, respectively. These reactions are considered reversible and can be subdivided into the forward R1a and R2a reactions and into the reversed reactions R1b and R2b, respectively. The equilibrium constants (*K*) of these reactions in a particular matrix are calculated by means of Formula (F1) for the formation of GAA and Orn (R1, *K*_gaa_) and by Formula (F2) for the formation of hArg and Orn (R2, *K*_harg_). Formulae (F1) and (F2) are based on the law of mass action and require knowledge of the equilibrium concentrations of the amino acids, which participate in the reactions. The ratio of *K*_gaa_ to *K*_harg_, i.e., *K*_gaa_/*K*_harg_ = *K*_AGAT_, describes the state of the equilibrium of the AGAT-catalyzed formation of GAA relative to hArg. In Formulae (F1), (F2) and (F3), the equilibrium concentrations of the amino acids are set in square brackets.

R1 $${\text{Arg }} + {\text{ Gly}} \rightleftarrows {\text{GAA }} + {\text{ Orn}}$$

R1a $${\text{Arg }} + {\text{ Gly}} \to {\text{GAA }} + {\text{ Orn}}$$

R1b $${\text{GAA }} + {\text{ Orn}} \to {\text{Arg }} + {\text{ Gly}}$$

R2 $${\text{Arg }} + {\text{ Lys}} \rightleftharpoons {\text{hArg }} + {\text{ Orn}}$$

R2a $${\text{Arg }} + {\text{ Lys}} \to {\text{hArg }} + {\text{ Orn}}$$

R2b $${\text{hArg }} + {\text{ Orn}} \to {\text{Arg }} + {\text{ Lys}}$$

F1 $$K_{{{\text{gaa}}}} = \, \left( { \, \left[ {{\text{GAA}}} \right] \, \times \, \left[ {{\text{Orn}}} \right] \, } \right) \, / \, \left( { \, \left[ {{\text{Arg}}} \right] \, \times \, \left[ {{\text{Gly}}} \right] \, } \right)$$

F2 $$K_{{{\text{harg}}}} = \, \left( { \, \left[ {{\text{hArg}}} \right] \, \times \, \left[ {{\text{Orn}}} \right] \, } \right) \, / \, \left( { \, \left[ {{\text{Arg}}} \right] \, \times \, \left[ {{\text{Lys}}} \right] \, } \right)$$

F3 $$K_{{{\text{AGAT}}}} = K_{{{\text{gaa}}}} /K_{{{\text{harg}}}} = \left( { \, \left[ {{\text{GAA}}} \right] \, / \, \left[ {{\text{hArg}}} \right] \, } \right) \, \times \, \left[ {{\text{Lys}}} \right] \, / \, \left[ {{\text{Gly}}} \right]$$

In vitro, the average equilibrium constant *K*_AGAT_ was determined to be about 1 for purified AGAT from hog kidney. This value was observed by using high initial concentrations of Arg and Gly (range, 1–10 mM), of Orn and GAA (range, 1–5 mM), and a long incubation time of 4 h at 40 °C (Ratner and Rochovansky 1956). The above-mentioned Arg and Gly concentrations are of the order of the *K*_M_ values of human kidney AGAT (Gross et al. 1986). The reaction rate of hArg as the amidine group donor (R2b) was reported to be six times lower than the reaction with Arg (R2a). In the study by Ratner and Rochovansky, the constant *K*_harg_ has not been reported (Ratner and Rochovansky 1956). These authors found that Orn was a product inhibitor of AGAT (Ratner and Rochovansky 1956).

The intracellular concentrations of amino acids are generally unknown. Intracellular amino acid concentrations are assumed to be in equilibrium with their extra-cellular concentrations such as in plasma and erythrocytes. The plasma and serum concentrations of the AGAT-reaction products GAA and hArg in healthy humans are in the lower µM-range (Atzler et al. 2016; Hanff et al. 2018, 2019a, 2019b; Kayacelebi et al. 2015, 2017). On the other hand, the plasma and serum concentrations of the AGAT substrates, Arg, Gly, Lys and Orn, in healthy humans are about two orders of magnitude higher compared to those of GAA and hArg. Based on the circulating concentrations of Arg, Lys, Gly, Orn, GAA and hArg in healthy humans, the *K*_gaa_ and *K*_harg_ values are expected to be far below 1 each, and that of *K*_AGAT_ about 1.

Reversible reactions can temporarily be disturbed by adding substrates/products at concentrations higher than those present at equilibrium. After a certain period, the original equilibrium is re-established. In vivo, intake of amino acids by the meal, as free amino acids and as proteins, results in temporary increases of the concentrations of circulating amino acids from exogenous sources. Despite repeated disturbances in circulating amino acids concentrations during normal day life, circulating amino acids are generally assumed to be at a relatively stable steady state. Much greater deviations from steady-state concentrations of amino acids would occur when particular amino acids would be introduced in the body in large amounts, for instance, by intravenous infusion. As an example, in the so-called L-arginine test, Arg is infused at a rate of 0.5 g (2.9 mmol) L-arginine per kg body weight for 30 min. In such cases, very high supra-physiological concentrations of L-arginine are reached in the blood, and the previous steady state is re-established several hours after stopping the infusion (Kayacelebi et al. 2015). Changes in circulating Arg concentrations are accompanied with changes in circulating concentrations of metabolites of Arg including free asymmetric dimethylarginine (ADMA) and hArg. ADMA and hArg are formed intracellularly in different metabolic pathways of Arg. Free ADMA is formed by the posttranslational dimethylation of Arg residues in numerous proteins catalyzed by protein-arginine methyl transferase (PRMT) and their concomitant proteolysis (Tsikas 2021). At present, it is not known whether free Arg is dimethylated to ADMA. It can be expected that part of the infused Arg is utilized in protein synthesis already during the L-arginine test (Kayacelebi et al. 2015), thus forming proteins which are subsequently dimethylated on Arg residues. The parallel formation of ADMA and hArg during Arg infusion (Kayacelebi et al. 2015) indicates that different Arg-involving processes are concerned due to the supply of large amounts of Arg. Analogous effects are seen in rats, when hArg is intra-peritoneally injected in high amounts (Günes et al. 2017). In this animal model, studies on tissue amino acids biochemically closely related to Arg indicated great temporary changes in tissue concentrations of several amino acids (Tsikas and Redfors 2022). The equilibria constants of AGAT-involving pathways in the rat organs differed greatly and changed by several orders of magnitude in the investigated organs in dependence on the hArg dose and the experimental time (Tsikas and Redfors 2022).

Based on the above-mentioned observations, we wanted to know whether the concentrations of circulating amino acids are in principal useful to determine equilibrium constants in vivo, and to study effects of administered amino acids such as Arg and hArg or drugs such a metformin. To investigate this issue, we chose the AGAT-catalyzed reactions (R1) and (R2) (Scheme 1). We measured the concentrations of Arg, Lys, Gly, Orn, GAA and hArg in serum samples of patients with Becker muscular dystrophy (BMD) before and after ingestion of the antidiabetic drug metformin and L-citrulline (Cit) alone and their combination each for six weeks (Hafner et al. 2016; Hanff et al. 2018). Metformin increases AMP-activated protein kinase activity in skeletal muscle of subjects with type 2 diabetes (Musi et al. 2002). Both metformin and Cit, the precursor of Arg, were found to be beneficial to the BMD patients (Hafner et al. 2016). By using the serum concentrations measured at three different time points of that study, we calculated the equilibrium constants *K*_gaa_ and *K*_harg_. The usefulness and potential limitations of this approach are discussed in this article.

## Methods

### Patients and supplementation

**Scheme 2:**
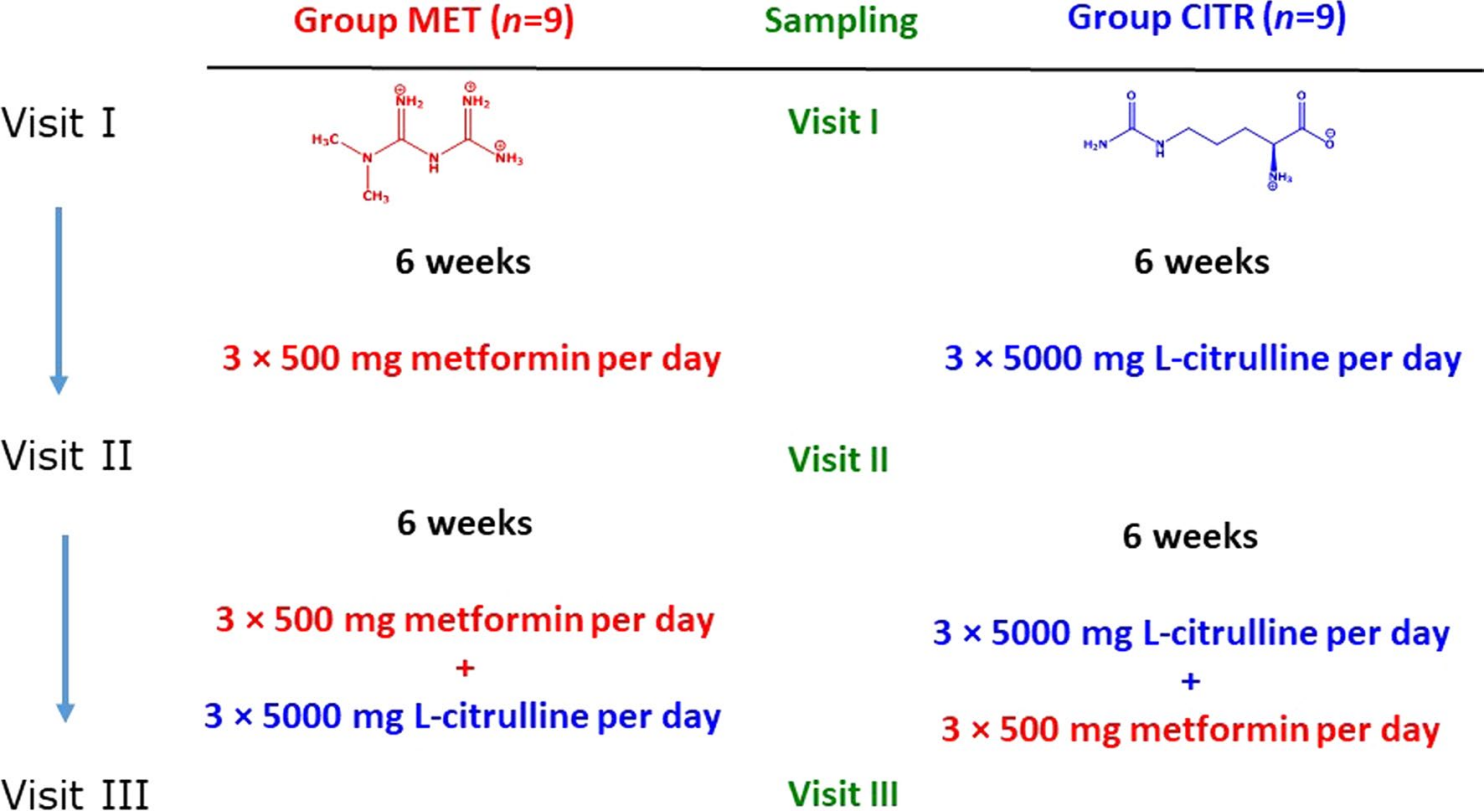
Study design on the effects of metformin and L-citrulline (each for six weeks) and their combination (for six weeks) in patients with Becker muscular dystrophy (BMD)

The underlying clinical study was approved by the local Ethics Committee and National Swiss Drug Agency, and was reported previously in detail (Hafner et al. 2016). All study visits were performed at the University of Basel Children’s Hospital (UKBB). Exclusion criteria were intake of supplementary Arg, Cit and metformin within the last three months. All patients of the study were male and did not differ with respect to their age or stature (Hafner et al. 2016). The Becker muscular dystrophy (BMD) patients were treated daily for six weeks either with 3 × 500 mg metformin (Sandoz Pharmaceuticals AG, Rotkreuz, Switzerland) (MET group) or with 3 × 5 g L-citrulline (L-citrulline drinking solution; Selectchemie Zuerich, Switzerland) (CITR group) (Scheme 2). Subsequently, patients ingested daily at the same time for a period of 6 weeks 3 × 500 mg metformin + 3 × 5 g L-citrulline. Blood samples were collected immediately prior to the first treatment (baseline; Visit I, day 0), immediately before starting the combined treatment (Visit II, week 6) and at the end of the combined medication (Visit III, week 12).

### Measurement of serum amino acids and metformin

**Scheme 3:**
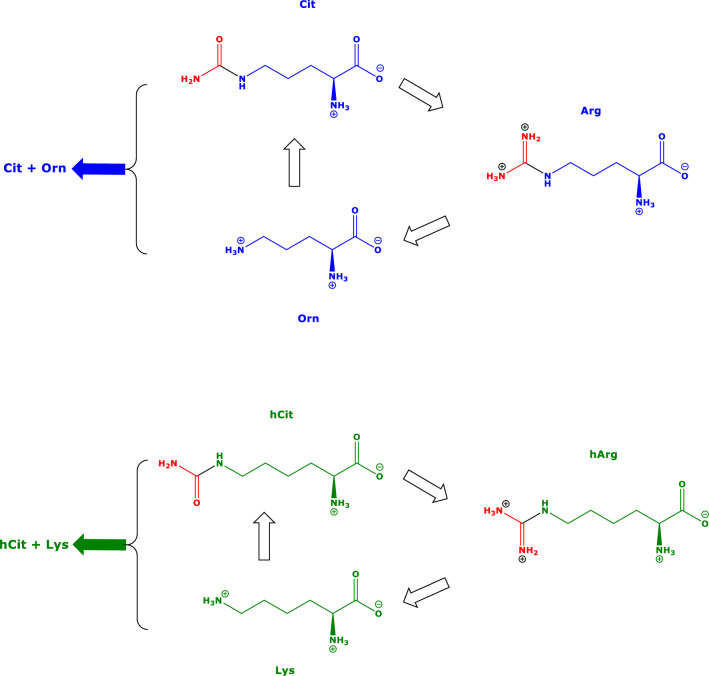
Simplified schematic of the urea cycles of Arg (upper panel) and hArg (low panel). The GC–MS method used in the present study cannot discriminate between Cit and Orn, nor between hCit and Lys, and provides the sum of the concentrations of Cit and Orn (Cit + Orn) and the sum of the concentrations of hCit and Lys (hCit + Lys) (Hanff et al. 2019b)

The concentrations of Arg, Lys, Gly, hArg, GAA, Orn, Cit (Hanff et al. 2018) and metformin (Baskal et al. 2022a) were determined in previously collected patients serum samples (Hafner et al. 2016) by gas chromatography-mass spectrometry (GC–MS) methods. As the GC–MS method for amino acids measures the sum of Orn and Cit (Hanff et al. 2019b), the equilibrium constants were calculated using the summed concentration of Orn and Cit (Orn + Cit) (Scheme 3). In analogy, the GC–MS method measures the sum of homocitrulline (hCit) and Lys (Hanff et al. 2019b), and the equilibrium constants were calculated using the summed concentration of hCit and Lys (hCit + Lys) (Scheme 3). The concentration of hCit in serum is much lower than the serum Lys concentration. The values of *K*_gaa_ and *K*_harg_ were calculated using Formulae (F1a) and (F2a), respectively. All GC–MS analyses were performed on a single-quadrupole mass spectrometer model ISQ directly interfaced with a Trace 1310 series gas chromatograph equipped with an autosampler AS 1310 (all from ThermoFisher; Dreieich, Germany).

F1a $$K_{{{\text{gaa}}}} = \left( { \, \left[ {{\text{GAA}}} \right] \, \times \, \left[ {{\text{Orn}} + {\text{Cit}}} \right] \, } \right) \, / \, \left( { \, \left[ {{\text{Arg}}} \right] \, \times \, \left[ {{\text{Gly}}} \right] \, } \right)$$

F2a $$K_{{{\text{harg}}}} = \, \left( { \, \left[ {{\text{hArg}}} \right] \, \times \, \left[ {{\text{Orn}} + {\text{Cit}}} \right] \, } \right) \, / \, \left( { \, \left[ {{\text{Arg}}} \right] \, \times \, \left[ {{\text{Lys}} + {\text{hCit}}} \right] \, } \right)$$

F3a $$K_{{{\text{AGAT}}}} = K_{{{\text{gaa}}}} /K_{{{\text{harg}}}} = \left( { \, \left[ {{\text{GAA}}} \right] \, / \, \left[ {{\text{hArg}}} \right] \, } \right) \, \times \, \left( { \, \left[ {{\text{Lys}} + {\text{hCit}}} \right] \, } \right) \, / \, \left[ {{\text{Gly}}} \right]$$

### Statistical analyses and data presentation

Statistical analyses were performed using GraphPad Prism 7.0 for Windows (GraphPad Software, San Diego, CA, USA). D’Agostino and Pearson omnibus K2 test was used to test for normality. The serum concentration of few amino acids was not distributed normally. For the sake of simplicity, data are presented as mean ± standard deviation (sd), and correlations were performed after Spearman. Two-tailed *P* values ≤ 0.05 were considered statistically significant. Differences within the MET and CITR groups with respect to the equilibrium constants were tested using one-way ANOVA with Tukey’s multiple comparisons test for the individual visits.

## Results

In the BMD patients, we have investigated the effects of ingestion of the antidiabetic drug metformin, of the non-proteinogenic amino acid Cit and of their combination each for six weeks (Hafner et al. 2016; Hanff et al. 2018; Baskal et al. 2022a). Metformin reduced the concentrations of hArg and GAA both in serum and in urine, suggesting inhibition of both AGAT-catalyzed reactions by metformin (Hanff et al. 2018). Instead, Cit supplementation alone increased the circulating and the urinary concentrations of hArg and GAA, presumably by increasing the bioavailability of Arg, the substrate of AGAT. The combined use of metformin and Cit has different effects than the single drugs (Hanff et al. 2018). Based on these previous observations, we thought that the above-mentioned study should be useful to investigate, whether the concentrations of amino acids in the circulation may be useful to determine equilibria constants of reactions that take place within cells and to monitor effects of drugs. This approach was previously proven useful to study AGAT-catalyzed reactions in main organs of anesthetized rats treated with hArg (Tsikas and Redfors 2022).

The concentrations of the reactants of the AGAT-catalyzed reactions, i.e., Gly, GAA, Orn + Cit, Lys + hCit, Arg and hArg, were measured simultaneously in the serum samples of the patients of the MET and CITR groups at Visit I (baseline, no metformin, no L-citrulline), Visit II (metformin or L-citrulline) and Visit III (metformin and L-citrulline) (Scheme 2). The measured serum concentrations of the above-mentioned amino acids are summarized in Table 1.

**Table 1:** Serum concentrations (µM) of Gly, GAA, Orn + Cit, Lys + hCit, Arg and hArg in the patients of the metformin (MET) and citrulline (CITR) groups at Visit I (VI), Visit II (VII) and Visit III (VIII)

Group	Gly			GAA			Orn + Cit			Lys + hCit			Arg			hArg		
	VI	VII	VIII	VI	VII	VIII	VI	VII	VIII	VI	VII	VIII	VI	VII	VIII	VI	VII	VIII
MET	252	207	202	1.64	0.69	1.23	63	49.2	115	178	149	113	107	87.5	130	0.66	0.39	0.86
MET	216	208	202	1.58	n.a	1.42	92.2	73.8	117	181	180	151	91.5	90.4	105	1.36	0.97	1.9
MET	288	259	268	2.82	2.27	3.58	76.3	57.5	227	245	209	189	155	136	227	1.36	0.98	1.8
MET	285	289	279	1.93	1.38	1.81	90.3	73.7	107	352	323	210	197	162	227	1.7	1.15	1.8
MET	249	211	219	2.09	1.64	1.73	98.9	90	106	248	218	215	154	117	143	2.12	1.31	2.21
MET	317	253	273	2.38	1.14	3.07	101	67.3	139	198	215	165	136	107	166	1.1	1.79	2.23
MET	223	250	236	2.52	0.80	2.03	97.9	73.7	127	254	258	209	127	129	110	2.4	1.8	2.01
MET	245	286	247	2.02	2.36	2.91	66.1	76.8	133	198	262	203	115	135	183	1.81	1.7	1.87
MET	292	219	218	1.81	1.09	1.48	118	60.1	85.9	277	158	166	152	93.3	120	1.03	0.87	1.35
MEAN	263	242	238	2.09	1.42	2.14	89.3	69.1	129	237	219	180	137	118	157	1.50	1.22	1.78
SD	34	33	30	0.41	0.63	0.84	17.8	12.1	40	55.9	55.3	34.1	31.6	25.2	47.1	0.55	0.48	0.43
CITR	290	210	185	2.24	3.12	2.82	82.8	182	264	206	124	121	133	216	179	1.52	1.52	1.25
CITR	230	216	256	1.76	2.27	1.72	64.1	91	132	185	138	195	125	150	145	1.43	1.54	2.13
CITR	277	274	270	1.83	2.1	1.13	51.1	92.1	83.1	201	196	196	133	166	141	0.76	2.39	1.49
CITR	237	307	271	1.84	2.01	1.66	59	126	87.2	210	243	204	130	201	139	1.42	3.15	2.61
CITR	312	227	316	2.46	3.74	8.46	83.5	224	452	184	133	204	92.1	135	271	1.57	2.92	3.19
CITR	374	333	370	2.29	3.99	1.5	76.9	184	116	241	160	209	166	270	180	1.58	1.58	1.07
CITR	216	218	215	1.71	3.77	3.58	78.1	277	222	231	227	235	110	243	278	1.67	3.18	3.86
CITR	283	241	251	2.65	2.2	1.4	87.6	131	118	229	237	173	140	214	148	1.08	2.02	1.66
CITR	225	298	211	1.87	2.35	4.63	54.7	83.7	231	182	195	134	113	132	287	1.07	0.75	1.78
MEAN	272	258	261	2.07	2.84	2.99	70.9	154	190	208	184	186	127	192	196	1.34	2.12	2.12
SD	51	45.8	56.6	0.34	0.81	2.36	13.7	66.9	119	22.1	46.5	36.8	20.9	48.9	63.6	0.30	0.85	0.94

*SD* standard deviation, *n.a* not available

At baseline, the serum concentrations of Gly, GAA, Orn + Cit, Lys + hCit, Arg and hArg were of the same order of magnitude as measured by us and others in serum samples of adult persons. The greatest differences were observed for Orn + Cit and Arg in the CITR group. The mean percentage changes of the serum concentrations of hArg and GAA were as follows. Supplementation of untreated BMD patients with Cit (i.e., CITR group) increased the serum hArg and GAA concentrations. Add-on supplementation with metformin did not change the serum hArg concentrations (± 0%), but increased slightly the serum GAA concentrations (+ 7.2%). The final hArg and GAA concentrations increased by 58.2% and 44%, respectively. Supplementation of untreated BMD patients with metformin (i.e., MET group) decreased the serum hArg and GAA concentrations. Add-on supplementation with Cit increased the serum hArg (+ 37.4%) and GAA (30.1%) concentrations. The final hArg and GAA concentrations increased by 18.7% and 2.4%, respectively.

**Fig. 1:**
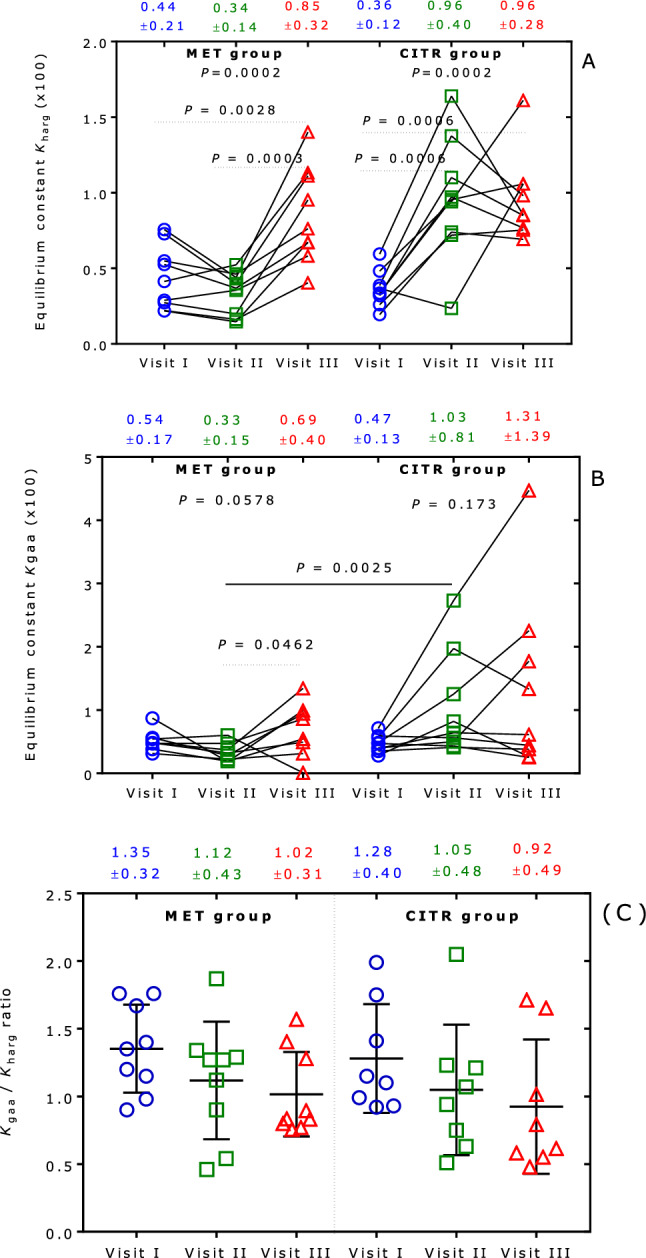
**A** Equilibrium constant *K*_harg_ of the reaction Arg + Lys $\rightleftarrows$ hArg + Orn. One-way ANOVA revealed statistical significances in the MET (*P* = 0.0002) and CITR (*P* = 0.0002) groups. Tukey’s multiple comparisons test: *P* = 0.0028 between Visit I and Visit III; *P* = 0.0003 in the MET group; each *P* = 0.0006 between Visit I and Visit II, and Visit I and Visit II in the CITR group. **B** Equilibrium constant *K*_gaa_ of the reaction Arg + Gly $\rightleftarrows$ GAA + Orn; one-way ANOVA revealed not statistical significance in the MET (*P* = 0.0587) and CITR (*P* = 0.173) groups; Tukey’s multiple comparisons test between Visit II and Visit III in the MET group: *P* = 0.0462. Unpaired t test (Mann–Whitney) revealed statistical significance between the Visits II of the MET and CITR groups (*P* = 0.0025). (**C**) *K*_gaa_/*K*_harg_ ratio in the MET and CITR groups of the study at Visits I, II and III. Data in (**C**) are shown as mean ± sd; one-way ANOVA revealed not statistical significance in the MET (*P* = 0.1495) and CITR (*P* = 0.1041) groups. Data on the top of each figure are presented as mean ± sd

The calculated *K*_harg_, *K*_gaa_ and *K*_gaa_/*K*_harg_ values are listed in Table 2 and shown in Fig. 1. In the MET group, the *K*_harg_ value was determined to be 0.0044 ± 0.0021 at Visit I (baseline), 0.0034 ± 0.0014 at Visit II and 0.0085 ± 0.0032 at Visit III (*P* = 0.0028 for VI vs. VIII; *P* = 0.0003 for VII vs. VIII). In the CITR group, the *K*_harg_ value was 0.0036 ± 0.0012 at baseline (VI), 0.0096 ± 0.0040 at VII and 0.0096 ± 0.0028 at VIII (*P* = 0.0006 for VI vs. VII; *P* = 0.0006 for VI vs. VIII; Fig. 1A). In the MET group, the *K*_gaa_ value was determined to 0.0054 ± 0.0017 at Visit I, 0.0033 ± 0.0015 at Visit II and 0.0069 ± 0.0040 at Visit III. In the CITR group, the *K*_gaa_ value was 0.0047 ± 0.0013 at Visit I, 0.0103 ± 0.0081 at Visit II and 0.0131 ± 0.0139 at Visit III (Fig. 1B). The *K*_gaa_/*K*_harg_ values in the two study groups are shown in Fig. 1C. The ratio ranged between about 0.5 and 2.0.

**Table 2:** Equilibrium constants *K*_harg_, *K*_gaa_ and *K*_gaa_/*K*_harg_ at Visits I, II and III

Group	Visit I			Visit II			Visit III		
	*K*_harg_	*K*_gaa_	*K*_gaa_/*K*_harg_	*K*_harg_	*K*_gaa_	*K*_gaa_/*K*_harg_	*K*_harg_	*K*_gaa_	*K*_gaa_/*K*_harg_
MET	0.0022	0.0038	1.76	0.0015	0.0019	1.27	0.0067	0.0054	0.80
MET	0.0076	0.0074	0.97	0.0044	n.a	n.a	0.0140	0.0078	0.56
MET	0.0027	0.0048	1.76	0.0020	0.0037	1.87	0.0095	0.0134	1.40
MET	0.0022	0.0031	1.40	0.0016	0.0022	1.34	0.0040	0.0031	0.76
MET	0.0055	0.0054	0.98	0.0046	0.0060	1.29	0.0076	0.0059	0.77
MET	0.0041	0.0056	1.35	0.0052	0.0028	0.54	0.0113	0.0094	0.83
MET	0.0073	0.0087	1.20	0.0040	0.0018	0.46	0.0111	0.0099	0.89
MET	0.0053	0.0047	0.90	0.0037	0.0047	1.27	0.0067	0.0086	1.28
MET	0.0029	0.0048	1.67	0.0035	0.0032	0.90	0.0058	0.0049	0.84
MEAN	0.0044	0.0054	1.33	0.0034	0.0033	1.12	0.0085	0.0076	0.90
SD	0.0021	0.0017	0.34	0.0014	0.0015	0.46	0.0032	0.0031	0.27
CITR	0.0046	0.0048	1.05	0.0103	0.0125	1.21	0.0152	0.0225	1.48
CITR	0.0040	0.0039	0.99	0.0068	0.0064	0.94	0.0099	0.0061	0.62
CITR	0.0053	0.0047	1.75	0.0068	0.0043	0.63	0.0045	0.0025	0.55
CITR	0.0031	0.0035	1.15	0.0081	0.0041	0.51	0.0080	0.0038	0.48
CITR	0.0077	0.0071	0.92	0.0364	0.0273	0.75	0.0261	0.0447	1.71
CITR	0.0030	0.0028	0.93	0.0067	0.0082	1.21	0.0033	0.0026	0.79
CITR	0.0051	0.0056	1.10	0.0160	0.0197	1.23	0.0131	0.0133	1.01
CITR	0.0030	0.0059	1.99	0.0052	0.0056	1.07	0.0077	0.0044	0.58
CITR	0.0028	0.0040	1.41	0.0024	0.0050	2.05	0.0107	0.0177	1.65
MEAN	0.0043	0.0047	1.25	0.0110	0.0103	1.07	0.0109	0.0131	0.98
SD	0.0016	0.0013	0.38	0.0103	0.0082	0.45	0.0068	0.0139	0.50
ALL	0.0044	0.0050	1.29	0.0072	0.0070	1.09	0.0097	0.0103	0.94
	0.0018	0.0015	0.36	0.0081	0.0069	0.45	0.0053	0.0102	0.39

*SD* standard deviation, *n.a*. not available

Under consideration of all data (*n* = 54), the *K*_harg_ value amounted to 0.0071 ± 0.0060 (range, 0.0015 to 0.0364), the *K*_gaa_ value 0.0075 ± 0.0073 (range, 0.0018 to 0.0447) and the *K*_harg_/*K*_gaa_ ratio 1.11 ± 0.42 (range, 0.46 to 2.05). The reported *K*_gaa_ value of 1.1 in vitro (Ratner and Rochovansky 1956) is much higher than the *K*_gaa_ value observed in our in vivo study, presumably due to very high concentrations and the long incubation time used in the in vitro study.

As both study groups ingested the two drugs in the last six weeks of the study (Scheme 2), we tested for correlations between the serum concentrations of hArg or GAA with the serum concentrations of metformin or Cit + Orn at Visit III in the collapsed MET and CITR groups. Serum hArg concentration did not correlate after Spearman with the serum metformin concentration (*r*_S_ = 0.287, *P* = 0.2481, *n* = 19). Serum GAA concentration did correlate with the serum metformin concentration (*r*_S_ = 0.7401, *P* = 0.0004, *n* = 19). Neither hArg nor GAA did correlate with the Cit + Orn concentration in the serum.

We also tested for correlations between the serum concentrations of the amino acids at the three visits for the collapsed data of the MET and CITR groups as well for the separate groups. Table 3 shows many, in part close correlations between substrates and products of the two AGAT-catalyzed reactions investigated in the present work, both at baseline and after drug ingestion. These correlations support the general assumption that amino acids stay in equilibrium in the body.

**Table 3:** Spearman correlation coefficients (*P* < 0.05) between the indicated pairs of serum concentrations of amino acids involved in the AGAT pathway in the patients measured at Visits I, II and III

Visit or group	Correlated pair	Correlation coefficient *r*	*P* value
Visit I	Lys vs. Arg	0.839	0.00001
	Lys vs. Orn	0.499	0.0347
	Gly vs. GAA	0.496	0.0366
	Gly vs. Arg	0.484	0.0418
Visit II	Orn vs. GAA	0.752	0.00075
	Orn vs. Arg	0.750	0.00034
	GAA vs. Arg	0.688	0.00292
	Orn vs. hArg	0.669	0.00239
	Arg vs. hArg	0.570	0.01353
	Gly vs. Lys	0.478	0.0449
Visit III	GAA vs. Orn	0.809	0.00005
	GAA vs. Arg	0.743	0.0004
	Orn vs. Arg	0.645	0.0038
	GAA vs. hArg	0.511	0.0303
	Lys vs. hArg	0.511	0.0304
	Lys vs. Gly	0.489	0.0395
MET group	Lys II vs. Gly II	0.7818	0.0105
	Lys I vs. Arg I	0.7660	0.0128
	GAA II vs. Arg II	0.7607	0.0438
	GAA III vs. hArg III	0.6485	0.0490
CITR group	Arg III vs. GAA III	0.90476	0.0046
	Arg III vs. Orn III	0.80952	0.0218
	Gly I vs. GAA I	0.73810	0.0458

## Discussion

In the present work, we investigated two reactions that are catalyzed by the same enzyme, i.e., arginine:glycine amidinotransferase (AGAT). This enzyme transfers the amidine group of amino acids serving as donors to the amino groups of certain amino acids serving as acceptors. AGAT has a broad spectrum of guanidine compounds as substrates (Humm et al. 1997a, b). Main substrates are Arg, a guanidino amino acid, Gly and Lys, whereas main AGAT products are GAA, hArg and Orn (Scheme 1). Orn is also an inhibitor of AGAT activity (Ratner and Rochovansky 1956; Sipilä 1980). We calculated the equilibria constants of the two main AGAT-catalyzed reactions by using measured concentrations of amino acids participating in these reactions. Previously, we found that the equilibria constants of AGAT-catalyzed reactions can be determined by using tissue concentrations of relevant amino acids in rats administered with high amounts of hArg; this study indicated the principal applicability of such an approach in vivo (Tsikas and Redfors 2022). The present study provides proof for the utility of this approach for circulating amino acids being reactants in AGAT-catalyzed reactions. In the in vivo study, untreated BMD patients ingested the antidiabetic drug metformin, the amino acid Cit (a precursor of Arg) or their combination for six weeks. Expectedly, the administration of Cit disturbed greatly the steady-state equilibria of the AGAT reactions as expressed by changes in the serum amino acid concentrations and in the equilibrium constants. Knowledge of circulating concentrations of amino acids provides important information about the amino acid homeostasis in a biological system. In the case of AGAT, which uses two amino acids as substrates and generates two amino acids or metabolites, knowledge of equilibria constants provides additional useful information, as they include the effects of all reactants.

Metformin is a pleiotropic drug (Graham et al. 2011; Gormsen et al. 2016; Song et al. 2021). It is a biguanidine, a chemically and metabolically inert compound, and has the potential to disturb directly AGAT-catalyzed reactions. Metformin seems to inhibit AGAT activity competitively, since add-on supplementation of Cit increased AGAT activity. The concentration of supplemented metformin in human blood is of the order of 10 µM. In tissue, however, such as in small intestine and kidney (Jeong and Jusko 2021), metformin may reach much higher concentrations than in plasma, high enough to compete with L-arginine in the active center of AGAT, thus reducing the formation of hArg and GAA. An inhibitory action of metformin on AGAT activity is supported by observations that the guanidino compounds creatine and GAA inhibit AGAT activity when supplied at high amounts (Edison et al. 2007; Taes et al. 2008; da Silva et al. 2014; Ostojic et al. 2016). In contrast, infusion of Arg or Cit, but not Gly, markedly increased renal GAA production in the rat (Edison et al. 2007), suggesting that AGAT activity crucially depends on Arg. In the MET group of our study, we observed a decrease in the serum Arg concentration at Visit II compared to Visit I. A considerable decrease in plasma Arg concentration of about 20% has been reported for patients with advanced type 2 diabetes within a few weeks after starting with metformin administration (Top et al. 2022), indicating immediate metformin-induced changes in Arg-involving pathways in the diabetic patients.

Supplementary Cit has a therapeutic potential as a source of Arg in health and diseases associated with Arg deficiency (Schwedhelm et al. 2007; Rashid et al. 2020). Supplementary Cit is preferred to supplementary Arg due to its favorable properties in the gastrointestinal tract including restricted metabolization. In adults, oral doses of several grams of Cit per day are required to increase circulating Arg concentrations. Our present and previous (Hanff et al. 2018) observations suggest that the AGAT pathway is influenced differently by supplemented metformin and Cit. Administration of Cit alone exerts opposite effects on the AGAT activity compared to metformin. hArg and Lys are closely interrelated metabolites in humans (Bollenbach et al. 2019) and in rats (Baskal et al. 2022b). In the AGAT pathway, Lys is the second substrate needed to produce hArg. Cit administration increases hArg and GAA formation most likely by increasing the bioavailability of Arg within AGAT-expressing cells. Add-on supplementation of metformin to Cit seems to “freeze” the activity of AGAT, while add-on supplementation of Cit to metformin seems to “re-activate” AGAT to produce hArg and GAA. In our study, we found a decrease of serum Cit concentration upon metformin supplementation. Such an effect of metformin has been reported in type 2 diabetes patients (Breier et al. 2017). From a therapeutic point of view, Cit seems to be a preferred drug to increase hArg synthesis. The Cit dose we used in the study is as effective as the hArg synthesis capacity of one kidney of healthy humans (Hanff et al. 2019a). The mechanisms by which hArg exerts its favorable health effects are not known, but they are likely to be largely independent of NO (Tsikas et al. 2018). Very recently, hArg administration was shown to inhibit atherogenesis by modulating *T* cell function (Nitz et al. 2022). For a recent review on hArg in health and disease, see (Tsikas 2022).

AGAT (Humm et al. 1997a, b) and dimethylarginine dimethylaminohydrolase (DDAH; EC 3.5.3.1) (Murray-Rust et al. 2001; see also Linsky and Fast 2010; Tsikas et al. 2022) share very similar catalytic mechanisms. DDAH hydrolyzes the guanidino compound asymmetric dimethylarginine (ADMA) to dimethylamine and Cit. AGAT and DDAH have the same catalytic triad and the catalytic process involves a nucleophilic attack of the sulfhydryl (SH) group of certain cysteine residues of the enzymes on the carbon atom of the guanidine groups of amino acids including Arg and its asymmetrically dimethylated metabolite ADMA. Such a mechanism seems to apply to other enzymes that accept guanidine compounds as substrates (Linsky and Fast 2010). Metformin is known not to undergo metabolism (Graham et al. 2011; Gormsen et al. 2016; Song et al. 2021). Metformin is likely to be a competitive inhibitor of AGAT, and presumably to a much lesser extent of DDAH. Yet, other mechanisms such as interactions of metformin, which is a permanently positively charged organic molecule, with amino acids residues of AGAT and DDAH are possible.

Our work is subject of general and methodological limitations. Amino acids circulate rapidly through the whole organism and their transport to intracellular compartments is presumably more rapid than their enzymatic metabolism and catabolism including protein synthesis (Gauthier-Coles et al. 2021). Although the circulating concentrations of many amino acids are closely correlated, they are not known for the majority of cell types and do not necessarily reflect the intracellular concentrations. Nevertheless, the concentrations of amino acids in human serum (present study), in human breast milk (Baskal et al. 2022c) and in rat tissues (Tsikas and Redfors 2022) are useful to estimate equilibria constants under various conditions including long-term pharmacological treatment. We showed this for metformin, a pleiotropic antidiabetic drug, for Cit, the physiological precursor of Arg, and for hArg, an AGAT-catalyzed metabolite of Arg and Lys, and a potential drug. A potential limitation of the present study is the relatively small number of patients, which was originally designed and performed as a proof-of-concept pilot trial (Hafner et al. 2016). A potential methodological limitation is the GC–MS method used to measure the concentrations of amino acids. It cannot discriminate between Orn and Cit, nor between Lys and hCit (Hanff et al. 2019b) and may have affected the calculated values of the equilibria constants. A modification of this GC–MS method was found to discriminate between Cit and Orn (Baskal et al. 2021) and could be useful in determining more accurately equilibria constants of AGAT-catalyzed reactions.

## Data Availability

Data are freely available to any researcher wishing to use them for non-commercial purposes.

## Abbreviations

*AGAT*: Arginine:glycine amidinotransferase
*BMD*: Becker muscular dystrophy
*Cit*: Citrulline
*CITR group*: Citrulline group
*GAA*: Guanidinoacetate
*GAMT*: Guanidinoacetate methyltransferase
*GC**–**MS*: Gas chromatography-mass spectrometry
*hArg*: Homoarginine
*hCit*: Homocitrulline
*MET group*: Metformin group
*Orn*: Ornithine
